# Supplementary figures and images for: The influence of mortality and socioeconomic status on risk and delayed rewards: a replication with British participants
Source: PeerJ. 2017 Jul 25;5:e3580. doi: 10.7717/peerj.3580 (PMC5530991; doi:10.7717/peerj.3580)

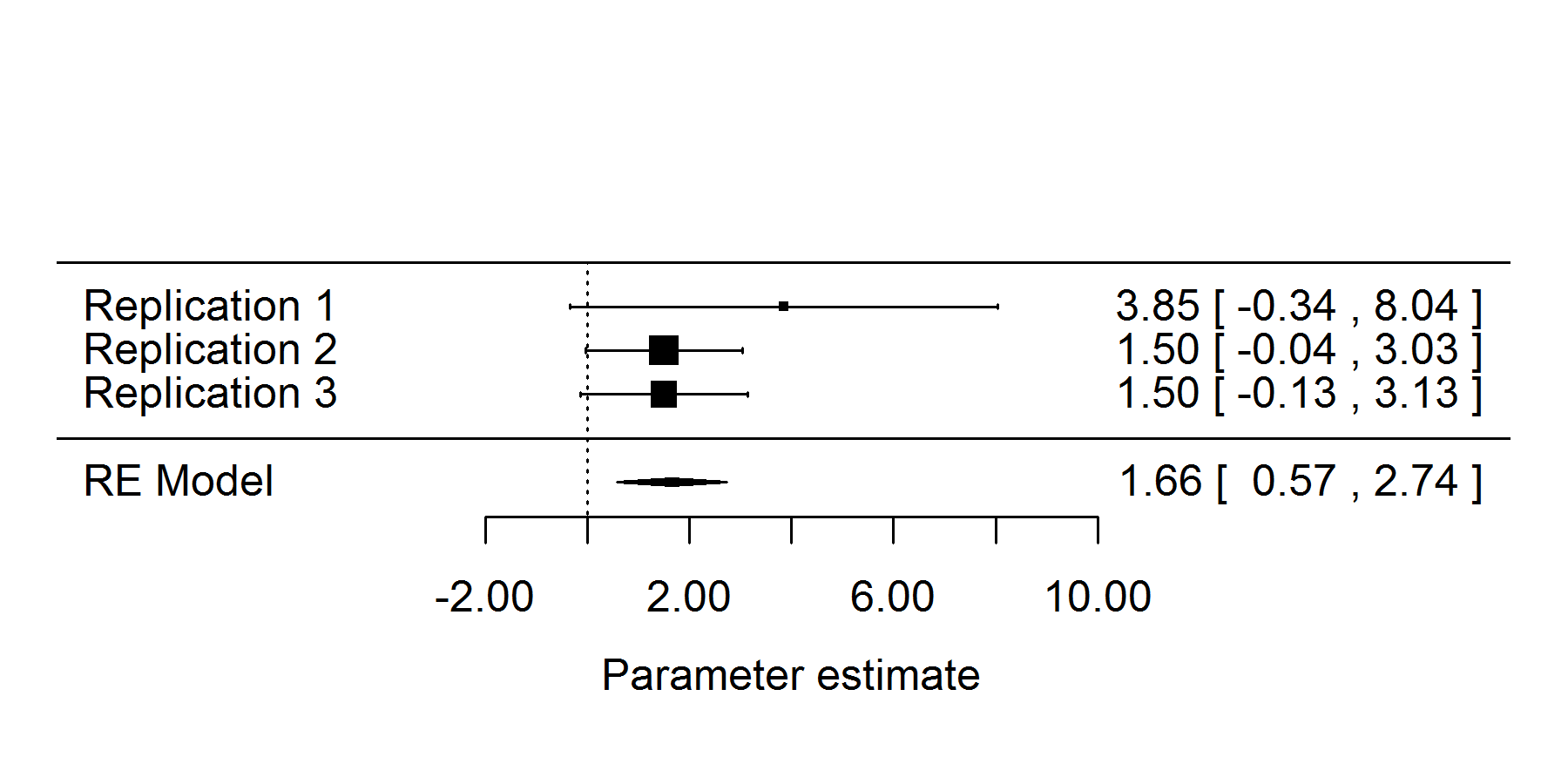

Supplement: Supplemental Information 1 — Forest plot for meta-analysis of the interaction between sex and condition in predicting delay discounting across our three replications. [file peerj-05-3580-s001.png]

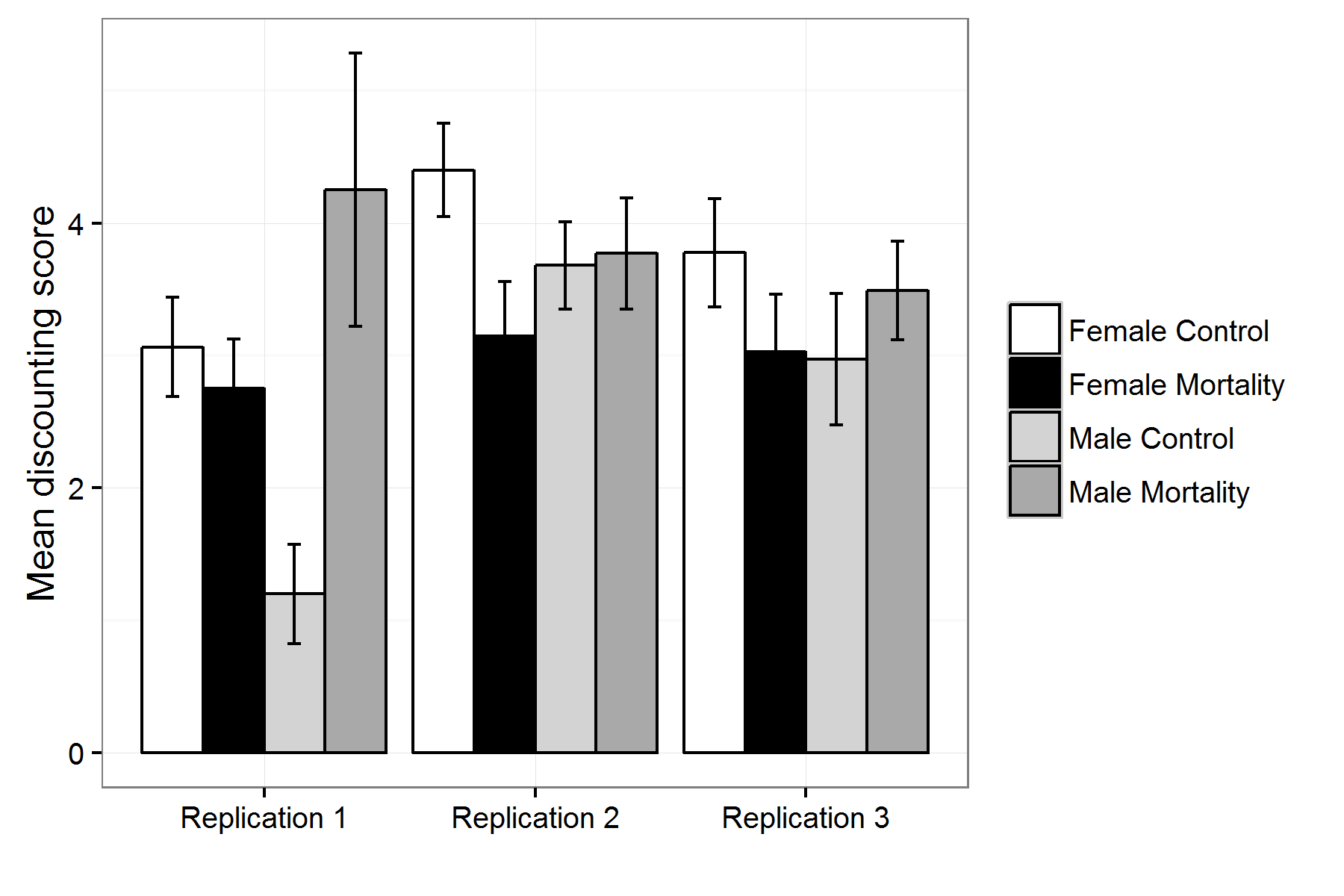

Supplement: Supplemental Information 2 — Mean discounting score broken down by replication, sex, and mortality-priming condition. Error bars represent one standard error. [file peerj-05-3580-s002.png]
