# Supplementary material for: The influence of mortality and socioeconomic status on risk and delayed rewards: a replication with British participants
Source: PeerJ. 2017 Jul 25;5:e3580. doi: 10.7717/peerj.3580 (PMC5530991; doi:10.7717/peerj.3580)
Supplement: Supplemental Information 2 [file peerj-05-3580-s004.docx]

**SUPPLEMENTARY ANALYSIS FOR PEPPER ET AL.: THE INFLUENCE OF MORTALITY AND SOCIOECONOMIC STATUS ON RISK AND DELAYED REWARDS: A REPLICATION WITH BRITISH PARTICIPANTS**

This supplementary analysis document is intended to report a more exhaustive set of results than we could reasonably present in the main paper. Section 1 provides descriptive statistics by condition for each replication. In section 2, we examine associations between the subjective socioeconomic status (SES) measures used in the main paper, and a more-objective measure of SES, the deprivation score based on the Index of Multiple Deprivation (IMD) score from the participant’s postcode. In section 3, we report additional analyses of the main datasets, in which sex and adult SES are added to the models as additional predictors and 3-way interactions are explored. In section 4, we report descriptive statistics for the responses to our adapted priming material by the participants in replication 3.

1. **DESCRIPTIVE STATISTICS BY CONDITION**

Tables S1, S2 and S3 provide descriptive statistics by condition for the three replications respectively.

|  | **Mean** | | **Standard deviation** | | **Median** | | **Range** | |
| --- | --- | --- | --- | --- | --- | --- | --- | --- |
|  | **Control** | **Prime** | **Control** | **Prime** | **Control** | **Prime** | **Control** | **Prime** |
| **Age** | 19.78 | 19.89 | 1.38 | 2.50 | 20 | 19 | 18 – 23 | 18 – 33 |
| **Risk** | 1.50 | 1.92 | 1.42 | 1.25 | 1.5 | 2 | 0 – 7 | 0 - 5 |
| **Discounting** | 2.81 | 2.92 | 2.07 | 2.14 | 2 | 3 | 0 – 7 | 0 – 7 |
| **Child SES** | 15.06 | 15.69 | 2.64 | 3.31 | 15 | 16.5 | 5 – 19 | 3 - 21 |
| **Adult SES** | 13.47 | 13.19 | 3.43 | 3.54 | 14 | 13 | 4 – 19 | 6 – 20 |

Table S1. Replication 1: Descriptive statistics by experimental condition.

|  | **Mean** | | **Standard deviation** | | **Median** | | **Range** | |
| --- | --- | --- | --- | --- | --- | --- | --- | --- |
|  | **Control** | **Prime** | **Control** | **Prime** | **Control** | **Prime** | **Control** | **Prime** |
| **Age** | 38.87 | 38.99 | 11.48 | 11.67 | 36 | 39 | 20-75 | 18-72 |
| **Risk** | 1.89 | 1.49 | 1.68 | 1.48 | 2 | 1 | 0-7 | 0-7 |
| **Discounting** | 4.00 | 3.46 | 2.30 | 2.44 | 4 | 4 | 0-7 | 0-7 |
| **Child SES** | 11.56 | 11.65 | 4.49 | 4.14 | 13 | 12 | 3-21 | 3-20 |
| **Adult SES** | 11.38 | 12.04 | 4.72 | 4.37 | 12 | 13 | 3-21 | 3-21 |

Table S2. Replication 2: Descriptive statistics by experimental condition.

|  | **Mean** | | **Standard deviation** | | **Median** | | **Range** | |
| --- | --- | --- | --- | --- | --- | --- | --- | --- |
|  | **Control** | **Prime** | **Control** | **Prime** | **Control** | **Prime** | **Control** | **Prime** |
| **Age** | 36.74 | 35.90 | 12.42 | 11.49 | 33.5 | 33 | 19-71 | 18-72 |
| **Risk** | 1.76 | 2.14 | 1.76 | 1.91 | 2 | 2 | 0-7 | 0-7 |
| **Discounting** | 3.39 | 3.29 | 2.80 | 2.63 | 3 | 3 | 0-7 | 0-7 |
| **Child SES** | 11.57 | 11.86 | 4.28 | 3.75 | 12 | 12.5 | 3-21 | 3-21 |
| **Adult SES** | 12.58 | 12.34 | 4.32 | 4.44 | 13.5 | 13 | 3-21 | 4-21 |

Table S3. Replication 3: Descriptive statistics by experimental condition.

1. **DEPRIVATION SCORES & SUBJECTIVE SES**

Participants in all three experiments were asked for their home postcode, which was used to obtain a deprivation score based on the IMD, an objective area-based measure of socioeconomic status. This measure was not used in the original study by Griskevicius et al. (2011), but provided us with an additional, objective, measure of socioeconomic status. The associations between this deprivation score and the subjective measures for each replication are reported in Table S4.

| *Deprivation score* | | **Subjective child SES** | **Subjective adult SES** |
| --- | --- | --- | --- |
| **Replication 1** |  | **r = -0.40, p < 0.01** | r = 0.07, p = 0.58 |
| **Replication 2** |  | r = 0.10, p = 0.24 | **r = -0.22, p = 0.01** |
| **Replication 3** |  | r = 0.11, p = 0.23 | r = -0.08, p = 0.37 |

Table S4. Correlations between deprivation scores from the IMD of the participant’s postcode, and subjective adult and child SES measures.

There was a significant correlation between subjective childhood SES and the deprivation score for replication 1 (Table S4). This was not particularly surprising as this sample consisted of undergraduate students, and their home postcode was likely to be the postcode they grew up in, and hence reflective of their childhood SES. Thus, for replication 1, we reran the models examining the interaction between experimental condition and child SES, using deprivation score instead of subjective childhood SES. The interaction remained non-significant for both outcome variables (Tables S5 & S6).

| *Risk acceptance* | **F** | **P** | **B** | **SE(B)** |
| --- | --- | --- | --- | --- |
| **Condition (mortality)** | 0.94 | 0.34 | 0.40 | 0.42 |
| **Deprivation Score** | 1.11 | 0.30 | -0.02 | 0.02 |
| **Condition * Deprivation Score** | 0.51 | 0.48 | 0.02 | 0.02 |

Table S5. Replication 1: Results of the general linear model examining effects of priming condition and deprivation score from childhood postcode on risk acceptance. Df = 1, 63

| *Delay discounting* | **F** | **p** | **B** | **SE(B)** |
| --- | --- | --- | --- | --- |
| **Condition (mortality)** | 0.54 | 0.47 | -0.54 | 0.74 |
| **Deprivation Score** | 0.85 | 0.36 | -0.02 | 0.03 |
| **Condition * Deprivation Score** | 1.60 | 0.21 | 0.05 | 0.04 |

Table S6. Replication 1: Results of the general linear model examining effects of priming condition and deprivation score from childhood postcode on delay discounting. Df = 1, 63

1. **ADDITIONAL ANALYSES**

For each replication and outcome variable, we also ran models containing, in addition to condition and childhood SES, adult SES, sex, and interactions up to three-way. The results are reported in Tables S7 and S8 for replication 1, S9 and S10 for replication 2, and S11 and S12 for replication 3.

| *Risk acceptance* | **F** | **p** | **B** | **SE(B)** |
| --- | --- | --- | --- | --- |
| **Condition (mortality)** | 2.04 | 0.16 | 0.50 | 0.35 |
| **Sex (male)** | 0.34 | 0.56 | 0.69 | 1.18 |
| **Child SES** | 0.46 | 0.50 | -0.20 | 0.29 |
| **Adult SES** | 0.24 | 0.62 | -0.14 | 0.27 |
| **Condition*Child SES** | 1.22 | 0.27 | 0.41 | 0.37 |
| **Condition*Adult SES** | 0.83 | 0.37 | -0.33 | 0.36 |
| **Sex*Child SES** | 0.62 | 0.44 | 0.86 | 1.10 |
| **Sex*Adult SES** | 0.15 | 0.70 | 0.43 | 1.13 |
| **Sex*Condition** | 0.13 | 0.72 | -0.50 | 1.40 |
| **Sex*Child SES*Condition** | 0.53 | 0.47 | -0.94 | 1.28 |
| **Sex*Adult SES*Condition** | 0.00 | 0.96 | 0.08 | 1.86 |

Table S7. Replication 1: Results of the model with additional predictors and three-way interactions for risk acceptance. Df = 1, 60

| *Delay discounting* | **F** | **p** | **B** | **SE(B)** |
| --- | --- | --- | --- | --- |
| **Condition (mortality)** | 0.34 | 0.56 | -0.31 | 0.53 |
| **Sex (male)** | 1.50 | 0.22 | -2.20 | 1.80 |
| **Child SES** | 1.22 | 0.27 | 0.48 | 0.44 |
| **Adult SES** | 2.30 | 0.13 | -0.63 | 0.42 |
| **Condition*Child SES** | 0.42 | 0.52 | -0.36 | 0.56 |
| **Condition*Adult SES** | 1.44 | 0.23 | 0.66 | 0.55 |
| **Sex*Child SES** | 0.96 | 0.33 | -1.64 | 1.68 |
| **Sex*Adult SES** | 0.21 | 0.65 | 0.78 | 1.71 |
| **Sex*Condition** | 3.24 | 0.08 | 3.85 | 2.14 |
| **Sex*Child SES*Condition** | 0.72 | 0.40 | 1.66 | 1.95 |
| **Sex*Adult SES*Condition** | 0.90 | 0.35 | 2.70 | 2.84 |

Table S8. Replication 1: Results of the model with additional predictors and three-way interactions for delay discounting. Df = 1, 60

| *Risk acceptance* | **F** | **p** | **B** | **SE(B)** |
| --- | --- | --- | --- | --- |
| **Condition (mortality)** | 2.64 | 0.11 | -0.61 | 0.38 |
| **Sex (male)** | 0.85 | 0.36 | 0.32 | 0.34 |
| **Child SES** | 0.20 | 0.66 | 0.11 | 0.24 |
| **Adult SES** | 4.06 | 0.05 | 0.56 | 0.28 |
| **Condition*Child SES** | 0.52 | 0.47 | 0.29 | 0.40 |
| **Condition*Adult SES** | 3.77 | 0.05 | -0.78 | 0.40 |
| **Sex*Child SES** | 0.22 | 0.64 | 0.16 | 0.33 |
| **Sex*Adult SES** | 1.15 | 0.29 | -0.38 | 0.35 |
| **Sex*Condition** | 0.09 | 0.76 | 0.16 | 0.51 |
| **Sex*Child SES*Condition** | 0.54 | 0.46 | -0.39 | 0.54 |
| **Sex*Adult SES*Condition** | 3.66 | 0.06 | 1.07 | 0.56 |

Table S9. Replication 2: Results of the model with additional predictors and three-way interactions for risk acceptance. Df = 1, 147

| *Delay discounting* | **F** | **p** | **B** | **SE(B)** |
| --- | --- | --- | --- | --- |
| **Condition** **(mortality)** | 5.10 | 0.03 | -1.28 | 0.57 |
| **Sex (male)** | 1.91 | 0.17 | -0.71 | 0.52 |
| **Child SES** | 2.06 | 0.15 | -0.53 | 0.37 |
| **Adult SES** | 0.35 | 0.56 | 0.25 | 0.42 |
| **Condition*Child SES** | 1.73 | 0.19 | 0.80 | 0.61 |
| **Condition*Adult SES** | 0.00 | 0.97 | -0.03 | 0.60 |
| **Sex*Child SES** | 1.77 | 0.19 | 0.67 | 0.50 |
| **Sex*Adult SES** | 1.68 | 0.20 | -0.68 | 0.53 |
| **Sex*Condition** | 3.65 | 0.06 | 1.50 | 0.78 |
| **Sex*Child SES*Condition** | 0.53 | 0.47 | -0.59 | 0.81 |
| **Sex*Adult SES*Condition** | 0.03 | 0.86 | -0.15 | 0.84 |

Table S10. Replication 2: Results of the model with additional predictors and three-way interactions for delay discounting. Df = 1, 147

| *Risk acceptance* | **F** | **P** | **B** | **SE(B)** |
| --- | --- | --- | --- | --- |
| **Condition (mortality)** | 0.27 | 0.60 | 0.22 | 0.42 |
| **Sex (male)** | 0.74 | 0.39 | 0.36 | 0.42 |
| **Child SES** | 1.75 | 0.19 | 0.34 | 0.26 |
| **Adult SES** | 0.92 | 0.34 | 0.31 | 0.32 |
| **Condition*Child SES** | 0.52 | 0.47 | -0.30 | 0.41 |
| **Condition*Adult SES** | 0.00 | 0.96 | 0.02 | 0.45 |
| **Sex*Child SES** | 0.01 | 0.93 | -0.03 | 0.42 |
| **Sex*Adult SES** | 0.17 | 0.68 | 0.19 | 0.45 |
| **Sex*Condition** | 0.22 | 0.64 | 0.27 | 0.58 |
| **Sex*Child SES*Condition** | 0.24 | 0.62 | 0.30 | 0.60 |
| **Sex*Adult SES*Condition** | 0.25 | 0.62 | -0.31 | 0.61 |

Table S11. Replication 3: Results of the model with additional predictors and three-way interactions for risk acceptance. Df = 1, 150

| *Delay discounting* | **F** | **p** | **B** | **SE(B)** |
| --- | --- | --- | --- | --- |
| **Condition (mortality)** | 1.90 | 0.17 | -0.83 | 0.60 |
| **Sex (male)** | 2.99 | 0.09 | -1.05 | 0.61 |
| **Child SES** | 0.32 | 0.57 | -0.21 | 0.37 |
| **Adult SES** | 4.63 | 0.03 | 0.99 | 0.46 |
| **Condition*Child SES** | 0.32 | 0.57 | 0.33 | 0.59 |
| **Condition*Adult SES** | 0.01 | 0.92 | 0.07 | 0.66 |
| **Sex*Child SES** | 3.55 | 0.06 | 1.14 | 0.60 |
| **Sex*Adult SES** | 0.62 | 0.43 | -0.51 | 0.65 |
| **Sex*Condition** | 3.24 | 0.07 | 1.50 | 0.83 |
| **Sex*Child SES*Condition** | 1.21 | 0.27 | -0.96 | 0.87 |
| **Sex*Adult SES*Condition** | 0.00 | 0.95 | -0.06 | 0.88 |

Table S12. Replication 3: Results of the model with additional predictors and three-way interactions for delay discounting. Df = 1, 150

The only trend that can be seen across the three replications is for the interaction between sex and condition, which was marginally non-significant in each individual replication. However, we fitted a meta-analytic model to the results of all three replications and found that the interaction was significant across all three replications taken together (B = 1.95, s.e.(B) = 0.57, z = 3.40, p < 0.001; figure S1).


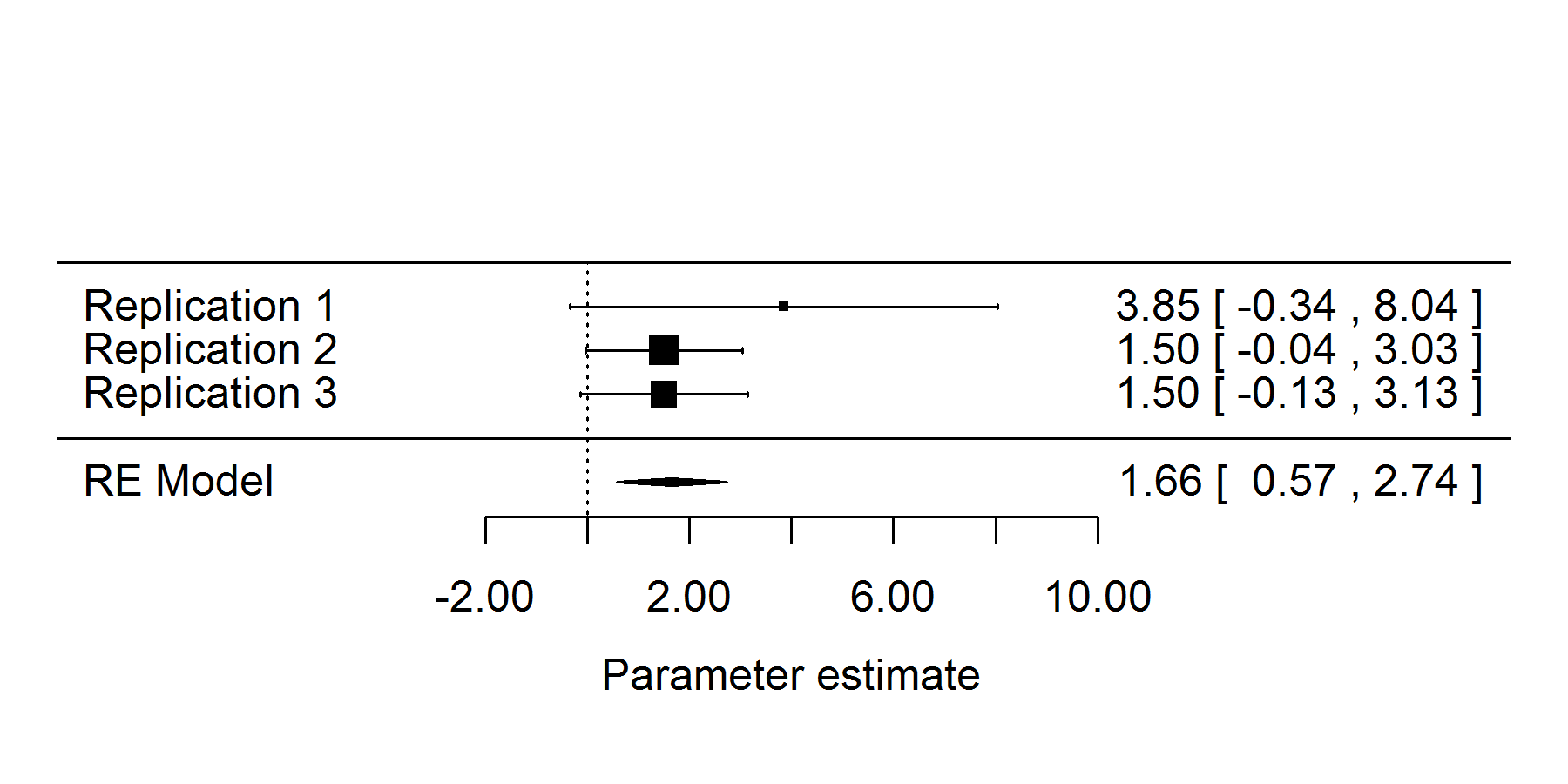


Figure S1. Forest plot for meta-analysis of the interaction between sex and condition in predicting delay discounting across our three replications.

The nature of the interaction across the three replications was that men tended to become more patient after priming, whereas women became less patient (figure S2). (Following the original study by Griskevicius et al (2011), we coded delay discounting such that a higher score indicates increasing patience.)


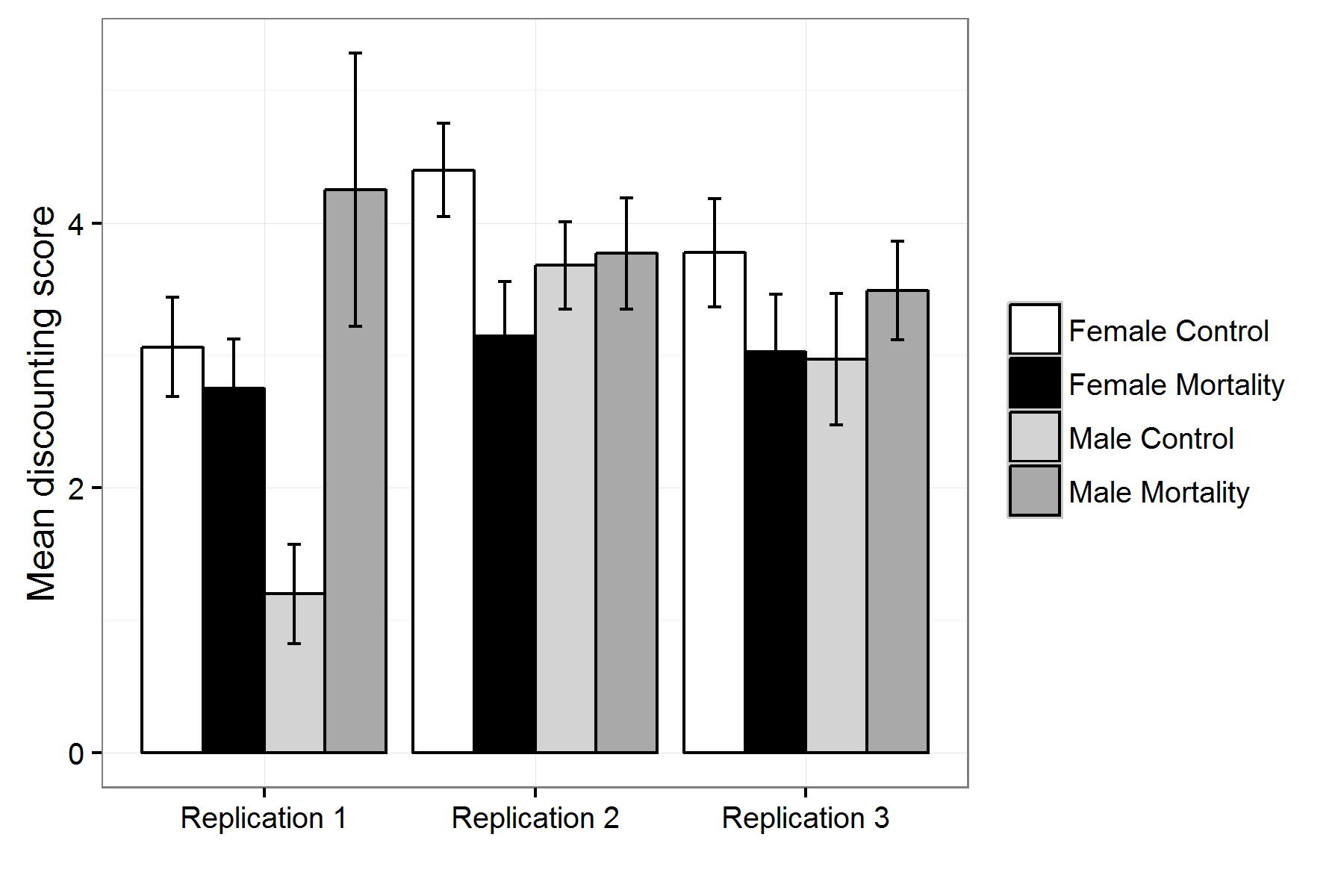


Figure S2. Mean discounting score broken down by replication, sex, and mortality-priming condition. Error bars represent one standard error.

1. **RESPONSES TO PRIMING MATERIAL IN REPLICATION 3**

To address the possibilities that we were unable to replicate the original findings of Griskevicius et al., (2011) in our first two replications either because we had altered the prime, or because the prime was less credible to British participants, we collected additional data during replication 3: After the outcome variables had been recorded, participants in the mortality-priming condition were also presented with the prime-piloting questions, described under Priming material (see main manuscript). The descriptive statistics for our participants’ responses to the priming material are below, in table S13.

| *Post-prime perceptions* | **Mean** | **Standard deviation** | **Median** | **Range** |
| --- | --- | --- | --- | --- |
| **Dangerous** | 4.87 | 1.53 | 5 | 1-7 |
| **Unsafe** | 4.81 | 1.32 | 5 | 1-7 |
| **Unpredictable** | 4.83 | 1.44 | 5 | 1-7 |
| **Uncertain** | 4.76 | 1.49 | 5 | 1-7 |
| **Aroused** | 3.95 | 1.49 | 4 | 1-7 |
| **Convincing** | 4.67 | 1.54 | 5 | 1-7 |

Table S13. Replication 3: Descriptive statistics for participant responses to prime piloting questions in replication 3 (see priming material, main manuscript for full piloting questions).

Exploratory analyses revealed that participants who felt that the world was unsafe after reading the prime were also subsequently less willing to accept risky options in the choice task. Participants who reported feeling that the world would become more dangerous after reading the prime also discounted future rewards less steeply. These post-priming perceptions did not have effects in interaction with child SES (see main manuscript table 4, and supplementary R script for more details).
